# UTE-T2^⁎^ Analysis of Diseased and Healthy Achilles Tendons and Correlation with Clinical Score: An In Vivo Preliminary Study

**DOI:** 10.1155/2017/2729807

**Published:** 2017-01-05

**Authors:** Yang Qiao, Hong-Yue Tao, Kui Ma, Zi-Ying Wu, Jian-Xun Qu, Shuang Chen

**Affiliations:** ^1^Department of Radiology, Huashan Hospital, Fudan University, Shanghai, China; ^2^Department of Sports Medicine, Huashan Hospital, Shanghai, China; ^3^GE Healthcare Applied Science Lab, Shanghai, China

## Abstract

*Objective*. To compare T2^⁎^ value of healthy and diseased Achilles tendons (AT) with a recently introduced three-dimensional ultrashort echo time (3D-UTE) sequence and analyze the correlation between T2^⁎^ value and clinical scores.* Methods*. Ten patients with symptomatic Achilles tendon and ten healthy volunteers were investigated with 3D-UTE sequence on a 3T magnetic resonance (MR) scanner. T2^⁎^ values of four regions in Achilles tendons were calculated. The clinical outcomes of patients were evaluated according to the American Orthopaedic Foot and Ankle Society (AOFAS) score and Achilles Tendon Rupture Score (ATRS). An independent sample *t*-test was used to compare the differences of T2^⁎^ value and clinical scores between two groups. The Pearson correlation coefficient between clinical scores and T2^⁎^ values was assessed.* Results*. The T2^⁎^ values of Achilles tendon were statistically significantly different between patients and volunteers. The Pearson correlation coefficients between T2^⁎^ and AOFAS or ATRS scores of patients were *r* = −0.733 and *r* = −0.634, respectively.* Conclusion*. The variability of T2^⁎^ in healthy and pathologic AT can be quantified by UTE-T2^⁎^. T2^⁎^ may be a promising marker to detect and diagnose AT tendinopathy. UTE-T2^⁎^ could give a precise guidance to clinical outcome.

## 1. Introduction

Tendinopathy, a syndrome with tendon pain, tenderness, and swelling that limited the tendon function, is one of the most common injures in both athletic and nonathletic populations [1, 2]. Nowadays, due to a higher involvement in sports, the prevalence of Achilles tendinopathy is increasing [3]. Although the exact etiology is still uncertain, it is supposed that repetitive overload and overuse are the major causes [4]. They may lead to irreversible degenerative changes of the Achilles tendon (AT), for instance, destruction and decrease of collagen fiber in extracellular matrix, increased vascularity, and altered cellularity [5–7]. Consequently, a precise way to detect Achilles tendinopathy is highly demanded.

As a noninvasive and credible diagnostic tool, magnetic resonance imaging (MRI) has been widely used to evaluate the pathological changes of AT. But highly organized AT with very short T2 appear dark on conventional MRI sequences, for only tissues with long T2 relaxation times could be visualized [8, 9]. Thus a short echo time sequence is needed to acquire signal from the AT. Three-dimensional ultrashort echo time (UTE) imaging, with an echo time as short as 0.05–0.5 ms, provided direct visualization and quantitative T2^⁎^-mapping for short-T2^⁎^ components [10–12]. Biochemical changes of early stage Achilles tendinopathy may affect T2^⁎^ values of AT and thus can be caught and quantified with UTE sequences [8].

Therefore, the aim of this study was to investigate the capability of quantitative 3D-UTE-T2^⁎^ in evaluating diseased AT and analyze the correlation between T2^⁎^ value and American Orthopaedic Foot and Ankle Society (AOFAS) score or Achilles tendon Total Rupture Score (ATRS). We hypothesize that the pathologic AT would show increased T2^⁎^ value while comparing with matched healthy samples and T2^⁎^ value may be correlated with clinical score.

## 2. Materials and Methods

### 2.1. Participants

The study was approved by the institutional review board of our hospital and all participants' informed consents were obtained. Ten patients (9 male/1 female, mean age 37.10 ± 8.60 years, BMI 23.00 ± 2.15 kg/m^2^) with pain or abnormalities in the AT and ten healthy volunteers matched for sex, age, and BMI (9 male/1 female, mean age 37.40 ± 10.61 years, BMI 23.94 ± 2.32 kg/m^2^) participated in the study. Participants were excluded if they had significant tendon rupture or any contraindication for MR.

### 2.2. MRI and Clinical Evaluation

All the participants were examined on a 3T MR scanner (Discovery 750, GE Healthcare, Waukesha, WI, USA) to get monoexponential calculation of T2^⁎^ in the human AT in vivo. As a quantitative 3D-UTE sequence, four echo times (TE = 0.032, 7.5, 20.5, and 28 ms) were acquired. The parameters were set as follows: sagittal orientation, FOV = 140 × 140 mm, slice thickness = 2.0 mm, flip angle = 18, and number of excitations (NEX) = 1. Fat-saturated proton-density weighted turbo-spin echo (PD-TSE) sequence was underwent to acquire morphological assessment with the parameters: sagittal orientation, TR 2843.0 ms, FOV 180 × 180 mm, slice thickness 2.0 mm, flip angle 142, and number of excitations (NEX) = 2.

For clinical evaluation, AOFAS scoring system and ATRS were used to evaluate the patients' clinical outcome (0–100 points, worst to best).

### 2.3. Imaging Analysis

**Figure 1:**
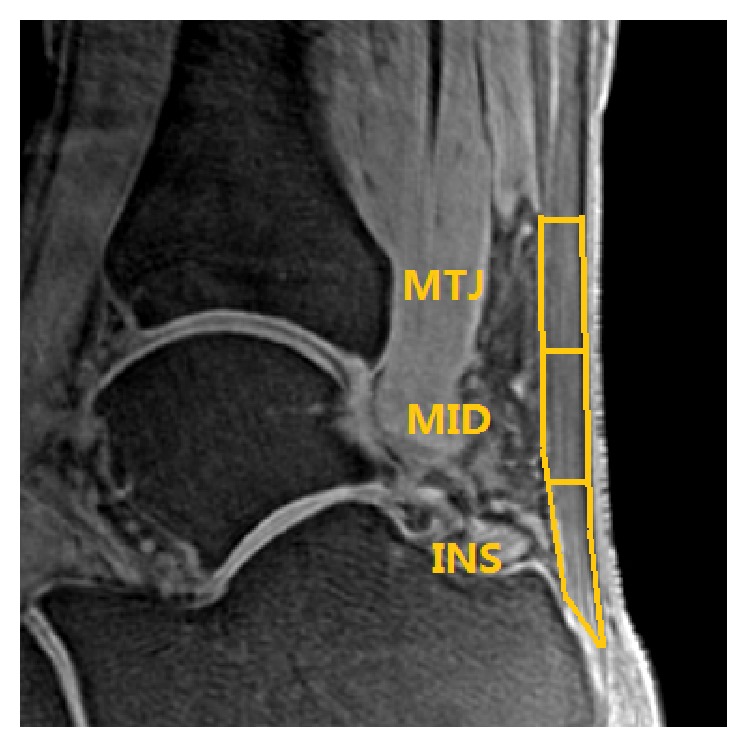
ROIs of Achilles tendon on sagittal UTE image acquired at TE = 0.032 ms. INS = insertion, MID = middle, MTJ = muscle-tendon junction; these three ROIs were equally divided according to the longitudinal length of Achilles tendon. The bulk ROI consisted of all the three ROIs.

**Figure 2:**
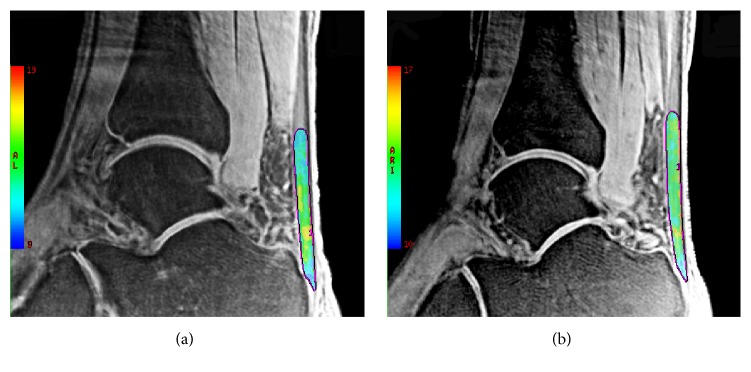
UTE T2^⁎^ mapping on diseased (a) and healthy (b) Achilles tendons of a 36-year-old male acquired at TE = 0.032 ms. Color scale represents T2^⁎^ values. The increase of T2^⁎^ values was observed in diseased tendon.

Images from the UTE-T2^⁎^ sequence were analyzed by software in the work station of GE. The AT was segmented and divided into three parts equally according to length: insertion (INS), middle (MID), and muscle-tendon junction (MTJ) (Figure 1). These three ROIs as well as all bulk of AT regions on each echo of UTE-T2^⁎^ images were drawn to get the mean MR signal. T2^⁎^ value of each region is calculated by fitting the acquired signal at different echo time to a single exponential decay model (Figure 2).

### 2.4. Statistical Analysis

All statistical analyses were performed in SPSS 20.0 (SPSS Institute, Chicago, IL, USA). An independent sample *t*-test was used to compare the differences of T2^⁎^ values between two groups. Pearson's correlation coefficient was used to analyze correlations between clinical scores and T2^⁎^ values of patients. The difference would be statistically significant if *P* value < 0.05.

## 3. Results

**Figure 3:**
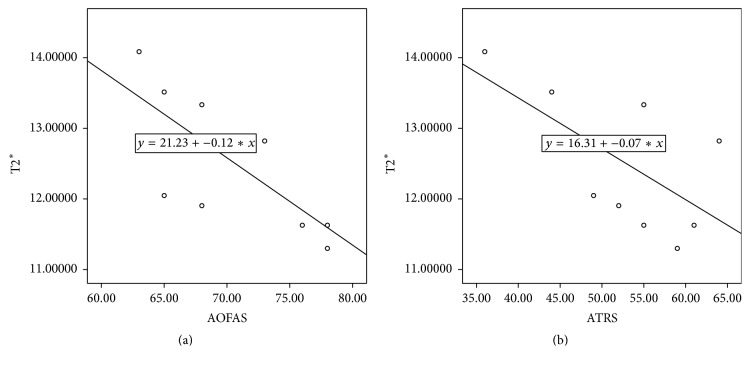
(a) A scatter plot of the bulk T2^⁎^ and AOFAS of ten patients. The Pearson correlation coefficient was *r* = −0.733 (*P* = 0.016). (b) A scatter plot of the bulk T2^⁎^ and ATRS of ten patients. The Pearson correlation coefficient was *r* = −0.634 (*P* = 0.049).

There were no obvious tendon tears on MRI for all patients. The mean T2^⁎^ value for bulk ROIs was significantly higher in patients than that in volunteers (12.508 ± 0.940 and 11.081 ± 0.297, *P* = 0.001) (Table 1). Separately, MTJ, MID, and INS regions of patients had statistically higher T2^⁎^ value compared with the matched regions of volunteers (MTJ: 11.977 ± 0.831 and 11.005 ± 0.581, *P* = 0.007, MID: 12.474 ± 1.261 and 11.124 ± 0.394, *P* = 0.008, and INS: 13.124 ± 0.943 and 11.084 ± 0.522, *P* = 0.000). The difference in INS region is greater than that in MID and MTJ. In patients, the mean AOFAS and ATRS were 70.6 ± 5.58 and 52.8 ± 8.27, respectively. The T2^⁎^ value for bulk region was negatively correlated with AOFAS as well as ATRS score (*r* = −0.733, *P* = 0.016, and *r* = −0.634, *P* = 0.049) (Figure 3).

In this study, T2^⁎^ relaxation time in pathologic and healthy AT was measured using UTE-T2^⁎^ sequence and a significant higher T2^⁎^ value was observed in all four regions of diseased AT. Besides, T2^⁎^ value of all bulk of AT in patients was found to be negatively correlated with AOFAS and ATRS score.

## 4. Discussion

UTE-T2^⁎^ mapping, a novel quantitative technique, could catch the short-T2^⁎^ relaxations from AT that are not well captured by standard T2 mapping [13]. In the early stages of Achilles tendinopathy, it is usually biochemical but not morphological changes that are found [14], which consist of destruction of collagen structure and increase of proteoglycan and water content [15]. UTE-T2^⁎^ mapping is sensitive to these changes; thus it could be a useful tool to detect tendon disease in an early stage [12]. The results of this study suggest that the variability of Achilles tendinopathy can be quantified by UTE-T2^⁎^. The increasing of T2^⁎^ value may due to disorganization of collagen structure and increasing of water content in tendons. What is more, the difference in INS region is greater. The reason could be that the enthesis is mostly involved in overuse injuries of AT [16, 17]. Gardin et al. [18] applied monoexponential calculation and showed a significant higher T2^⁎^ relaxation time in symptomatic tendons compared with control tendons. In a study by Juras et al., they compared mono- and biexponential T2^⁎^ analysis using variable-echo time sequence (vTE) and found that increased T2^⁎^ in all parts of diseased AT with monoexponential analysis [11]. Juras et al. also reported a similar finding with bicomponent quantitative 3D-UTE. They found significant differences between healthy (10.28 ± 2.28 ms) and degenerated AT (12.85 ± 1.87 ms) in the long component of T2^⁎^ [7]. While estimating the diagnostic value of T1 and T2^⁎^ relaxation times and off-resonance saturation ratio, Grosse et al. also observed statistical significant differences between the patients with tendinopathy and controls [19].

We applied monoexponential calculation of UTE-T2^⁎^. Juras et al. [11] found that the short component of T2^⁎^ reflects the changes of Achilles tendinopathy more accurately than the monoexponential T2^⁎^ [11]. However, owing to the longer scanning time and higher sensitivity to movements and the magic angle, biexponential calculation is more difficult to be applied clinically [18].

To the best of our knowledge, only a few studies analyzed the correlation between T2^⁎^ value and clinical score. Both AOFAS and ATRS scores are widely used in clinical practice and validated in many studies [20, 21]. They had general assessment of the AT situation. T2^⁎^ value of the bulk region in patients was correlated with AOFAS and ATRS score, which suggests that T2^⁎^ could give a precise guidance to clinical outcome of patients with Achilles tendinopathy. Juras et al. got a similar correlation between ATRS score and the monoexponential T2^⁎^ [11].

There are also some limitations in the study. Firstly, a small number of patients which would lead to increased statistical deviation. The patient cohort would be enlarged in our next study. Secondly, monoexponential calculation of T2^⁎^ reflects the mean value of all the components of relaxation time, which may lead to an underrate of T2^⁎^, especially in diseased tendons [7].

## 5. Conclusion

In conclusion, the differences between T2^⁎^ in healthy and pathologic tendons could be observed by UTE-T2^⁎^. As the preliminary patient data suggest, UTE-T2^⁎^ is an acute marker to detect AT tendinopathy in the early stage and it gives a precise guidance to clinical outcome. By further investigation in larger cohort of patients, different terms of follow-up after treatments are required to define the exact role of UTE-T2^⁎^ for monitoring the change of AT.

## Supplementary Material

SPSS software and Shapiro-Wilk method are used to test the distribution of samples, for the sample sizes are less than 2000. A normal distribution of our data was shown with the *P*-value > 0.05 each.

## Figures and Tables

**Table 1:** Comparison of T2^*∗*^ values between patients and volunteers. Mean (M) and standard deviation (SD) as well as *P* value were presented. The *P* value applied the significant difference in the four parts. Mean (M) and standard deviation (SD) of AOFAS and ATRS scores were also presented.

		Patients		Volunteers		*P* values
		M	SD	M	SD	
T2^*∗*^ values	Bulk	12.508	0.940	11.081	0.297	0.001
	MTJ	11.977	0.831	11.005	0.581	0.007
	MID	12.474	1.261	11.124	0.394	0.008
	INS	13.124	0.943	11.084	0.522	0.000
AOFAS score		70.6	5.58			
ATRS score		52.8	8.27			
