# Supplementary material for: UTE-T2⁎ Analysis of Diseased and Healthy Achilles Tendons and Correlation with Clinical Score: An In Vivo Preliminary Study
Source: Biomed Res Int. 2017 Jan 5;2017:2729807. doi: 10.1155/2017/2729807 (PMC5244000; doi:10.1155/2017/2729807)
Supplement: Supplementary file 1 — SPSS software and Shapiro-Wilk method are used to test the distribution of samples, for the sample sizes are less than 2000. A normal distribution of our data was shown with the P-value > 0.05 each. [file 2729807.f1.doc]

| **Tests of Normality** | | | | | | |
| --- | --- | --- | --- | --- | --- | --- |
|  | Kolmogorov-Smirnov^a^ | | | Shapiro-Wilk | | |
|  | Statistic | df | Sig. | Statistic | df | Sig. |
| bulk1 | .188 | 10 | .200^*^ | .935 | 10 | .494 |
| MTJ1 | .244 | 10 | .093 | .872 | 10 | .106 |
| MID1 | .169 | 10 | .200^*^ | .915 | 10 | .318 |
| INS1 | .258 | 10 | .059 | .905 | 10 | .250 |
| AOFAS | .179 | 10 | .200^*^ | .915 | 10 | .314 |
| ATRS | .161 | 10 | .200^*^ | .955 | 10 | .730 |
| bulk2 | .122 | 10 | .200^*^ | .982 | 10 | .974 |
| MTJ2 | .136 | 10 | .200^*^ | .929 | 10 | .434 |
| MID2 | .187 | 10 | .200^*^ | .915 | 10 | .318 |
| INS2 | .204 | 10 | .200^*^ | .921 | 10 | .365 |
| *. This is a lower bound of the true significance. | | | | | | |
| a. Lilliefors Significance Correction | | | | | | |
